# Catalase Deactivation Increases Dermatophyte Sensitivity to ROS Sources

**DOI:** 10.3390/jof10070476

**Published:** 2024-07-11

**Authors:** Sebastian Jusuf, Michael K. Mansour

**Affiliations:** 1 Department of Medicine, Harvard Medical School, Boston, MA 02115, USA; 2 Division of Infectious Diseases, Massachusetts General Hospital, Boston, MA 02114, USA

**Keywords:** dermatophyte, light, catalase, ROS, phototherapy

## Abstract

As the leading cause of fungal skin infections around the globe, dermatophytes are responsible for a multitude of skin ailments, ranging from athlete’s foot to ringworm. Due to the combination of its growing prevalence and antifungal misuse, antifungal-resistant dermatophyte strains like *Trichophyton indotineae* have begun to emerge, posing a significant global health risk. The emergence of these resistant dermatophytes highlights a critical need to identify alternative methods of treating dermatophyte infections. In our study, we utilized a 405 nm LED to establish that blue light can effectively inactivate catalase within a variety of both susceptible and resistant dermatophytes. Through this catalase inactivation process, light-treated dermatophytes were found to exhibit increased sensitivity to reactive oxygen species (ROS)-producing agents, improving the performance of antimicrobial agents such as H_2_O_2_ and amphotericin B. Our findings further demonstrate that light-induced catalase inactivation can inhibit the formation and polarized growth of hyphae from dermatophytes, suppressing biomass formation. Thus, by increasing ROS sensitization and inhibiting hyphal development, catalase-deactivating blue light offers a potential non-invasive and non-drug-reliant method of managing dermatophyte infections, opening new avenues for the potential treatment of these common infections in conjunction with existing treatments.

## 1. Introduction

Fungal skin infections, commonly referred to as superficial or cutaneous mycoses, are among the most common infections worldwide. As recently as 2016, fungal skin infections were ranked as the 4th most common disease in global incidence, impacting populations primarily within lower socioeconomic regions due to crowded living conditions and poorer hygiene [1]. While various different fungal species can trigger skin infections, most skin mycoses worldwide are predominantly caused by dermatophytes, a group of ascomycetous molds that encompass a range of different filamentous fungal genera, consisting of the genera *Trichophyton*, *Microsporum*, and *Epidermophyton* [2]. These dermatophytes are unique in their ability to digest and obtain nutrients from keratinized tissue like hair, skin, and nails and their adaptability to various environmental conditions [3,4,5].

In humans, dermatophytes are responsible for a variety of well-known highly contagious skin infections known as tinea, including athlete’s foot (tinea pedis), onychomycosis (tinea unguium), and jock itch (tinea cruris) [6]. Initially spreading throughout the environment in a unicellular and protected conidia form, dermatophytes can become metabolically active and form filamentous hyphae upon encountering favorable environmental conditions like keratin-rich skin or hair [7], leading to skin infection and irritation. Due to their ease of spread, dermatophyte infections (dermatophytosis) have become the most common type of superficial fungal infection worldwide, significantly impairing quality of life [8,9]. Together, these dermatophyte infections are estimated to constitute over half of all out-patient visits for fungal infections, inducing healthcare costs of over USD 800 million and a total economic burden of about USD 1.2 billion annually in the United States alone [10,11]. In recent years, however, a combination of socioeconomic problems, rising international travel, immigration from tropical regions, and increased contact with animal carriers has resulted in a significant increase in dermatophyte infections worldwide, highlighting concerns regarding the growing spread and prevalence of these infections [12,13].

As superficial or cutaneous mycosis, the treatment of most dermatophyte infections primarily consists of topically or systemically administered antifungal agents consisting of allylamines, azoles, and/or polyenes. For first-line treatments, dermatophyte infections are often treated with over-the-counter or prescription topical creams consisting of allylamines like terbinafine or azoles like itraconazole, inhibiting fungal growth through ergosterol biosynthesis disruption [14,15,16]. However, due to the growing prevalence of dermatophyte infections, the global consumption of antifungal agents like terbinafine is steadily increasing, especially among middle-income countries [17]. As the strains most responsible for human dermatophytosis, *Trichophyton rubrum* and *Trichophyton mentagrophytes* have already been found to be developing resistance mechanisms to antifungal agents like terbinafine through the use of membrane efflux pump overexpression, the production of drug-inactivating enzymes, and mutations in antifungal targets [18,19]. However, within the last few years, the emergence of *Trichophyton indotineae*, a recently discovered multidrug-resistant species of *Trichophyton* with reduced susceptibility to azole drugs and resistance to terbinafine, has also become a significant healthcare concern [20,21]. In countries like India, *T. indotineae* has overtaken *T. rubrum*, now representing the leading cause of dermatophytosis, and recent reports have indicated that the multidrug-resistant pathogen has begun spreading in Asia, Europe, and North America due to both travel and migration [22,23,24,25]. The growing proliferation of these multidrug-resistant dermatophytes is especially concerning due to their risk of becoming invasive, especially among immunocompromised and immunosuppressed individuals [26,27]. The emergence of these antifungal-resistant dermatophytes, coupled with increasing prevalence, highlights a critical need to identify alternative methods of treatment for dermatophyte infections that can not only improve the performance of existing antifungal agents but also provide a more effective and non-invasive method of treating these superficial skin infections.

Over the past few decades, phototherapy has emerged as a novel and non-invasive method of targeting antimicrobial infections. Currently, common applications of phototherapy for skin infections like herpes and acne are primarily constrained to photodynamic therapy, which relies on exogenous photosensitive reactive oxygen species (ROS)-generating agents like methylene blue to react with light and eliminate infections [28]. However, recent studies have discovered that specific wavelengths of blue light alone can exert a strong antimicrobial effect on various bacterial, fungal, and viral pathogens [29]. Investigations into the specific molecular interactions between light and pathogens revealed that certain molecules expressed naturally within microbes, such as porphyrins, are photosensitive, and continuous exposure to blue light can convert these photosensitive porphyrins into internal sources of ROS that can damage the interior of the pathogen [30]. In addition, studies have identified several specific photosensitive molecules and virulence factors, some broadly expressed across multiple bacterial and fungal strains, that can be degraded or deactivated by blue-light exposure. 

One such broad-spectrum target is the enzyme known as catalase, a commonly expressed H_2_O_2_-neutralizing antioxidant enzyme that is deactivated upon exposure to 405 nm blue light within bacterial and fungal strains, rendering the light-treated pathogens hypersensitive to ROS-producing agents like H_2_O_2_, specific antibiotics, and antifungals, as well as the host immune cells [31,32]. The photoinactivation of catalase has also been found to suppress the hyphae-forming abilities of the dimorphic fungal species *Candida albicans* through lipid metabolism disruption, reducing its potential virulence and immune evasion capabilities [33]. The photoinactivation of catalase by blue-light exposure represents an effective, non-invasive method of targeting and sensitizing a broad spectrum of pathogens. 

Since dermatophytes primarily invade through superficial or cutaneous skin infections, blue-light phototherapy is a non-invasive method of targeting and treating dermatophyte infections. The previous literature indicates that many pathogenic dermatophytes, including *T. rubrum*, *T. mentagrophytes*, *Microsporum gypseum*, and *Epidermophyton floccosum*, all express catalase activity to guard against oxidative stress [34,35]. Furthermore, *T. rubrum*’s resistance to antifungal compounds like undecanoic acid and acriflavine has been found to correspond to increased catalase expression [36,37]. Existing antifungal agents, such as itraconazole, terbinafine, and amphotericin B, have also been found to induce internal mitochondrial ROS production in fungal species, allowing for light-induced catalase deactivation to potentially synergize with conventional antifungal agents, improving their efficiency [38]. Based on these common attributes of dermatophytes, we hypothesize that catalase is a critical contributor to dermatophyte virulence, supporting hyphal growth and acting as a defensive mechanism against antimicrobial sources of ROS produced indirectly through antifungal sources. By utilizing the catalase-deactivating capabilities of 405 nm blue light, we believe that catalase photoinactivation can sensitize clinical dermatophyte isolates, including antifungal-resistant strains such as *T. indotineae*, to ROS-producing sources, providing a new potential avenue for the treatment of dermatophytes. 

## 2. Methods and Materials

### 2.1. Dermatophyte Strains and Culture

The strains used include *Trichophyton tonsurans* 10217^TM^ (ATCC, Manassas, VA, USA), *T. tonsurans* 10270^TM^ (ATCC), *T. rubrum* T2549631 (Massachusetts General Hospital (MGH), Boston, MA, USA), *T. rubrum* DI-23-182, *T. indotineae* DI-23-55, *T. indotineae* DI-23-56, *T. indotineae* DI-23-57, and *T. indotineae* DI-23-62. The *T. rubrum* DI-23-182 and *T. indotineae* strains were provided by Dr. Nathan P. Wiederhold from the University of Texas Health Science Center in San Antonio.

For culturing and dermatophyte isolation, the *Trichophyton* strains were streaked and grown on potato dextrose agar (DF0013-17-6, Fisher Scientific, Hampton, NH, USA) slants for 7 to 10 days at a temperature of 30 °C. Following incubation, spores were collected by adding 10 mL of sterile 1× Phosphate-Buffered Saline (PBS) and using a sterile inoculation loop to release conidia from colonies. Once the conidia fragments had been collected, the suspension was filtered through a 40 µm nylon Falcon cell strainer (08-771-1, Fisher Scientific) to filter out hyphal fragments. Once filtration was complete, the suspension was centrifuged and resuspended in 1 to 2 mL of sterile PBS to increase the overall conidia concentration. A LUNA-FL Dual Fluorescence Cell Counter (Logos Biosystems, Annandale, VA, USA) was used to confirm the removal of hyphal fragments and quantify the conidia concentration.

### 2.2. Blue-Light Device

Blue light was delivered through a 405 nm blue-light LED (M405L4, Thorlabs, Newton, NJ, USA) and focused to an area of 1 cm^2^ via a collimation adapter (SM2F32-A, Thorlabs). This device was provided by Dr. Ji-Xin Cheng from Boston University. A T-Cube LED driver (LEDD1B, Thorlabs) was used to adjust the fluence of the light. Blue light was delivered at a constant fluence of 200 mW/cm^2^ for all experiments. 

### 2.3. Catalase Assays

An Amplex Red Catalase Assay (A22180, ThermoFisher Scientific, Waltham, MA, USA) was used to measure the catalase activity within Trichophyton strains. To summarize, aliquots of conidia from individual strains were exposed to 60 J/cm^2^ of blue light and then diluted in 1× PBS to reach a final concentration ranging from 10^6^ to 10^7^ CFU/mL. Once treated, a 25 μL aliquot of the suspension was treated with 25 μL of a 40 μM H_2_O_2_ solution and incubated at room temperature within a 96-well plate. Following incubation, 50 μL of an Amplex Red and horseradish peroxidase mixture was added, and the solution was incubated at 37 °C for 30 min. Once complete, fluorescence measurements were collected at excitation/emission wavelengths of 560/590 nm. Measurements were performed in triplicate (*n* = 3). By comparing the fluorescence of the light-treated suspension to an untreated suspension and a negative PBS control, the reduction in catalase activity was determined. 

### 2.4. PrestoBlue and CFU Assays 

To determine the viability and metabolic activity of *Trichophyton* strains, assays were performed through a combination of CFU measurements and PrestoBlue Cell Viability Reagent (A13262, ThermoFisher Scientific). For CFU viability measurements, a 20 μL aliquot of 5 × 10^6^ conidia/mL was treated with 60 J/cm^2^ of blue light and then resuspended in 1980 μL of PBS. Dilutions were separated into 500 μL groups and then treated with varying concentrations of H_2_O_2_ and incubated at 30 °C for 2 h. Due to the differing sensitivity to H_2_O_2_ between dermatophyte strains, H_2_O_2_ concentrations that resulted in only minor to minimal antimicrobial activity were selected for each strain. After incubation, treatment groups were serially diluted by factors of 10, 100, and 1000 by combining 100 μL of each dilution with 900 μL of PBS. Once dilutions were complete, 10 μL aliquots of each dilution were plated on potato dextrose agar plates and fully evaporated before the plates were sealed with parafilm and incubated at 30 °C for up to 96 h. Upon the completion of incubation, the resulting colony number for each dilution was quantified. All dilutions were plated in triplicate (*n* = 3). 

For PrestoBlue assays, the *Trichophyton* suspension was aliquoted onto a glass cover slide and treated with varying dosages of blue light. The aliquots were then removed and resuspended in either PBS for H_2_O_2_ assays or MOPS-RPMI for antifungal assays to reach a final concentration between 1 and 3 × 10^6^ conidia/mL. For H_2_O_2_ assays, 100 μL of the dermatophyte PBS suspension was placed within a 96-well plate and treated with varying concentrations of H_2_O_2_ for 2 h at 30 °C. Following incubation, 100 μL of MOPS-RPMI and 22.2 μL of PrestoBlue reagent were added to each well. Once the reagent was added, the plate was incubated at 30 °C for up to 110 h. During this incubation period, the fluorescence of the PrestoBlue reagent was quantified at an excitation/emission of 560/590 nm. For antifungal assays, 200 μL of treated *Trichophyton* strains resuspended in MOPS-RPMI was treated with varying concentrations of amphotericin B (PHR1662, Sigma Aldrich, St. Louis, MO, USA) resuspended in DMSO (D8418, Sigma Aldrich). Once the antifungal had been added and adequately mixed, 22.2 μL of PrestoBlue reagent was added to each well. The plate was then incubated at 30 °C for up to 72 h, and fluorescence was quantified during incubation. Metabolic activity at specific time points could be determined by dividing the fluorescence of various treatment groups by the fluorescence intensity of the untreated group, revealing the reduction in viability of specific treatment groups. Each treatment group was analyzed in triplicate (*n* = 3). Imaging of the dermatophytes following the completion of the PrestoBlue viability assay was performed through a Nikon Eclipse TS100 inverted microscope (Nikon, Tokyo, Japan) under a magnification of 10× and captured through an Excelis HD camera (AU-600-HD, Unitron, Boston, MA, USA) via CaptaVision+ Version 2.4.1.10 (ACCU-SCOPE, Inc., Commack, NY, USA). 

### 2.5. Agar Invasion Assay 

To determine the impact of blue-light exposure on the hyphal development of dermatophytes and their potential invasion capabilities, a modified version of an agar invasion assay [39] was performed on potato dextrose agar. To examine the impact of hyphal growth, a 2 μL aliquot of 1 × 10^7^ conidia/mL *T. rubrum* T2549631 was treated with 60 J/cm^2^ of blue light and placed on the surface of a potato dextrose agar plate. Following incubation for 24 h at 30 °C, the agar surface containing the dermatophyte aliquot was treated with another 60 J/cm^2^ of blue light before continuing its 30 °C incubation for another 24 h. Once a total of 48 h had passed, the resulting dermatophyte growths were cut in half via a scalpel, extracted fully from the agar, and then placed horizontally on a Petri dish so the cross-section of the dermatophyte biomass and hyphal extensions were fully visible. Images of the hyphal growths were captured through a Nikon Eclipse TS100 inverted microscope under a magnification of 10× and processed via ImageJ 1.53q. Hyphal lengths in each condition were measured by individually measuring the hyphae from the extended tip to the base of the overall external biomass. 

When examining the overall difference in dermatophyte biomass formation caused by light treatment, both susceptible *T. rubrum* T2549631 and antifungal-resistant *T. indotineae* DI-23-57 were tested. For biomass studies, a 2 μL aliquot of 2.5 × 10^8^ conidia/mL was placed on potato dextrose agar and incubated at 30 °C for 48 h. Like previous invasion assays, light-treated groups received two 60 J/cm^2^ blue-light treatments during the incubation session, with one treatment occurring every 24 h. Once incubation was complete, the resulting dermatophyte biomass was bisected, extracted, and placed in a cross-section facing down on a Petri dish. The resulting biomass was imaged through a Nikon Eclipse TS100 inverted microscope under a magnification of 10× and processed via ImageJ. All biomass studies were performed in triplicate (*n* = 3). For the determination of agar invasion capabilities under both blue-light and hydrogen peroxide treatments, a 2 μL aliquot of 2.5 × 10^8^ conidia/mL was placed on potato dextrose agar, as previously established. For these samples, light treatment consisted of only one treatment application of 60 J/cm^2^ 405 nm light at the start of incubation. For H_2_O_2_ application, 10 μL of various concentrations of H_2_O_2_ was applied to the location at which the initial dermatophyte droplet was placed and allowed to fully evaporate before being incubated at 30 °C for 48 h. Following incubation, the resulting biomass was bisected, extracted, and imaged based on previously established methods.

### 2.6. Statistical Analysis

All statistical analysis and significance values were determined using a two-way ANOVA test via Student *t*-test analysis through PRISM 10.2.3 (GraphPad, San Diego, CA, USA). 

## 3. Results

### 3.1. Blue Light Reduces Catalase Activity in Dermatophytes

**Figure 1:**
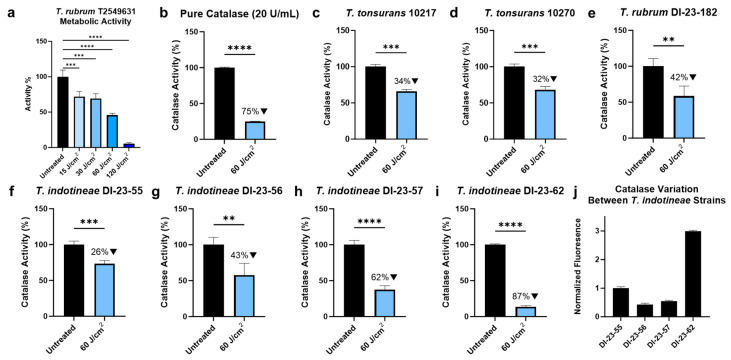
Blue-light treatment results in reduced catalase activity within dermatophytes. (**a**) Dermatophytes exhibit a dose-dependent reduction in metabolic activity based on the total amount of 405 nm blue light applied. When 60 J/cm^2^ of blue light is applied to (**b**) pure catalase samples, significant reductions in catalase activity are observed. When the same dosage is applied to the susceptible *Trichophyton* strains (**c**) *T. tonsurans* 10217 and (**d**) *T. tonsurans* 10270, a roughly 30% reduction in catalase activity is observed. For the resistant strains (**e**) *T. rubrum* DI-23-182, (**f**) *T. indotineae* DI-23-55, (**g**) *T. indotineae* DI-23-56, (**h**) *T. indotineae* DI-23-57, and (**i**) *T. indotineae* DI-23-62, treatment with 60 J/cm^2^ of blue light results in varying significant reductions in catalase activity. Interestingly, when examining the natural catalase activity present in untreated *T. indotineae* strains, (**j**) significant variations in catalase activity were observed, indicating that catalase activity is not uniform between strains. Statistical significance: ****: *p* < 0.0001, ***: *p* < 0.001, **: *p* < 0.01.

As one of the most abundant biological molecules expressed in organic life, catalase plays a significant role in a wide variety of organic processes, ranging from the neutralization of oxidative damage to lipid metabolism. To examine the potential effects of catalase-inactivating blue light on dermatophytes, various dosages of 405 nm light were applied to *T. rubrum* T2549631, and the overall change in metabolic activity was quantified through a PrestoBlue assay (Figure 1a). Based on the results, dermatophytes like *T. ruburm* T2549631 appear to exhibit a dose-dependent response to blue-light treatment, experiencing a 30% reduction in metabolic activity under 15 to 30 J/cm^2^ dosages, a 54.27% reduction under 60 J/cm^2^, and a 94.51% reduction under 120 J/cm^2^. Based on the dose responses and previous catalase inactivation studies, 60 J/cm^2^ was chosen as the standard dosage to apply to dermatophytes in light treatments. When validating the catalase-inactivating effects of 405 nm light, the treatment of pure catalase (Figure 1b) to 60 J/cm^2^ of 405 nm blue light was found to result in a 75.11% reduction in catalase activity, confirming catalase’s photosensitivity to blue-light exposure. When applying the same light treatment to wild-type susceptible dermatophyte strains *T. tonsurans* 10217 (Figure 1c) and 10270 (Figure 1d), catalase activity reductions of 34.28% and 31.93% were observed, respectively. While both susceptible strains exhibited similar decreases in catalase activity, additional testing on resistant dermatophyte strains found that the measured change in catalase activity can vary heavily between strains. However, significant reductions in activity were observed in all strains tested. In *T. rubrum* DI-23-182 (Figure 1e), a 41.53% reduction in catalase activity was observed. However, for *T. indotineae* DI-23-55 (Figure 1f), DI-23-56 (Figure 1g), DI-23-56 (Figure 1h), and DI-23-62 (Figure 1i), catalase activity was found to be reduced by 26.49%, 42.5%, 62.35%, and 86.61%, respectively. Given the large variation in catalase activity reduction observed between *T. indotineae* strains, an additional examination of the difference in catalase activity between strains was performed by normalizing the fluorescence signal associated with catalase activity for the untreated *T. indotineae* strains (Figure 1j). By normalizing the catalase activity fluorescence to the *T. indotineae* DI-23-55 strain, it was found that while strains DI-23-56 and DI-23-57 expressed roughly half as much catalase activity, *T. indotineae* DI-23-62 expressed significantly higher catalase activity, with a 3-fold increase in fluorescence-associated catalase activity. Based on catalase activity alone, significant variation in catalase activity, and therefore, natural catalase expression, exists between strains.

### 3.2. Blue Light Sensitizes Dermatophytes to Hydrogen Peroxide

**Figure 2:**
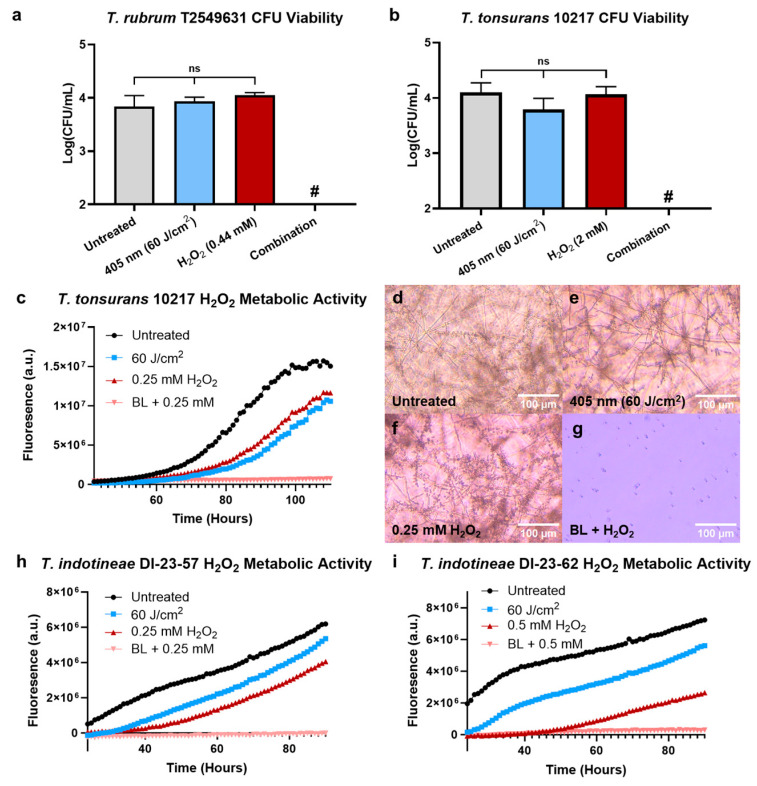
Treatment with blue light and H_2_O_2_ in combination reduces dermatophyte viability. By combining a set 60 J/cm^2^ dosage of blue light with low-concentration H_2_O_2_, CFU measurements reveal that greater than 99% reduction in dermatophyte colony formation can be observed in both (**a**) *T. rubrum* T2549631 and (**b**) *T. tonsurans* 10217, indicating increased dermatophyte sensitivity to ROS sources following light exposure. (**c**) Long-term PrestoBlue assays of *T. tonsurans* 10217 further confirm a complete reduction in the metabolic activity of light and H_2_O_2_-treated samples, and imaging reveals that while (**d**) untreated, (**e**) 405 nm treated, and (**f**) H_2_O_2_-treated groups exhibit strong filamentous proliferation, (**g**) combination-treated groups remain in an inactive and non-viable conidia form. Similar assays performed on the antifungal-resistant *T. indotineae* strains (**h**) DI-23-57 and (**i**) DI-23-62 exhibit similar patterns in the complete reduction in activity following the combination treatment. Abbreviations: BL = blue light. Statistical significance: ns = No Significance; # = Below Detection Limit.

The potential increase in ROS sensitization caused by light-induced catalase inactivation was explored through a combination of CFU measurements and additional PrestoBlue viability assays. The utilization of CFU quantification on *T. rubrum* T2549631 following individual and combined treatments of 60 J/cm^2^ of 405 nm light and sub-mM concentrations of hydrogen peroxide revealed that, while individual treatments of light and H_2_O_2_ had no impact on the CFU of *T. rubrum*, the combination of the two treatments resulted in a greater than 2 log reduction in CFU/mL (Figure 2a). Similar CFU tests performed on a less H_2_O_2_-sensitive species of *Trichophyton* in the form of *T. tonsurans* 10217 resulted in identical results, with no changes in viability occurring under individual treatments and nearly complete eradication observed under the combination treatment (Figure 2b). The eradication of dermatophytes was further validated through a long-term PrestoBlue assay, which showed that no significant fluorescence activity corresponding to metabolic activity was observed in *T. tonsurans* 10217 following combination treatment with 60 J/cm^2^ of 405 nm light and 0.25 mM of H_2_O_2_ exposure (Figure 2c). Imaging of *T. tonsurans* from this assay following 110 h of incubation additionally revealed that while significant hyphal formation and filamentation are observed within the untreated (Figure 2d), light-treated (Figure 2e), and H_2_O_2_-treated (Figure 2f) samples, individual dermatophyte conidia could still be observed within the combination-treated group (Figure 2g), with no significant hyphal formation detected. Following these results, additional tests were also performed on the antifungal-resistant *T. indotineae* strains DI-23-57 (Figure 2h) and DI-23-57 (Figure 2i). The resistant strains were found to display no significant metabolic activity following treatment with light and H_2_O_2_, mirroring the response of the susceptible strain to combination treatment and indicating that ROS sensitization occurs within both susceptible and resistant dermatophyte strains.

### 3.3. Blue Light Sensitizes Resistant Dermatophytes to ROS-Producing Antifungals

**Figure 3:**
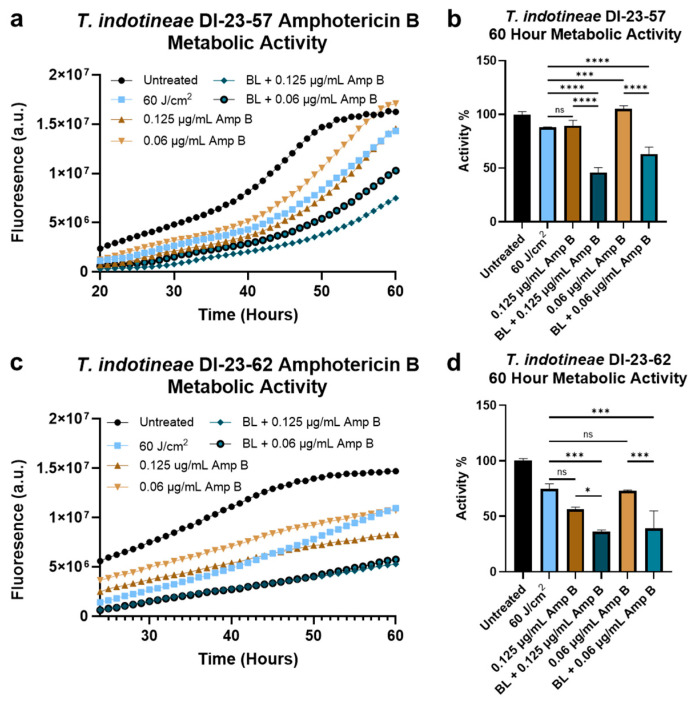
Blue-light treatment improves the performance of ROS-producing amphotericin B against antifungal-resistant dermatophytes. For *T. indotineae* DI-23-57, (**a**) metabolic activity assays and (**b**) calculated activity percentages at 60 h reveal a significant improvement in amphotericin B activity following exposure to 60 J/cm^2^ of 405 nm light. Similar results were observed in *T. indotineae* DI-23-62, where (**c**) metabolic activity assays and (**d**) calculated activity percentages reveal a significant improvement in the antifungal activity of 0.06 μg/mL amphotericin B following light treatment. Abbreviations: BL = blue light; Amp B = amphotericin B. Statistical significance: ****: *p* < 0.0001, ***: *p* < 0.001, *: *p* < 0.05, ns = No Significance.

After exploring the increased ROS sensitization of dermatophytes by H_2_O_2_ following blue-light exposure, attempts to synergize this phenomenon with existing antifungal agents were made. For these tests, amphotericin B was selected as the antifungal agent of choice due to the significant contributions that ROS formation has been found to play in the antifungal activity of amphotericin B [40,41]. Thus, 72 h PrestoBlue assays were performed on resistant strains of *T. indotineae* to observe any significant metabolic changes induced by amphotericin B in combination with blue light. For *T. indotineae* DI-23-57, the assay (Figure 3a) indicated the improved performance of 0.125 and 0.06 μg/mL amphotericin B when treated in combination with 60 J/cm^2^ of blue light. Further examination of the specific change in dermatophyte metabolic activity at the 60 h timepoint reveals that while treatments with light or amphotericin B individually retained, at most, a 10% reduction in activity, the combination treatment caused a significantly greater reduction, decreasing metabolic activity by 53.97% and 36.76% for light therapy in combination with 0.125 and 0.06 μg/mL amphotericin B, respectively (Figure 3b). For *T. indotineae* DI-23-62, the PrestoBlue assay (Figure 3c) found that while the strain was more sensitive to amphotericin B alone, the addition of light significantly improved the performance of 0.06 μg/mL amphotericin B. At the 60 h timepoint, while individual treatments of light or 0.06 μg/mL amphotericin B resulted in a roughly 25% reduction in metabolic activity, the combination of light and 0.06 μg/mL amphotericin B caused a 60.87% reduction in activity, greater than the individual treatment effects combined (Figure 3d). 

### 3.4. Blue Light Inhibits Hyphal Formation and Invasion Capabilities of Dermatophytes

**Figure 4:**
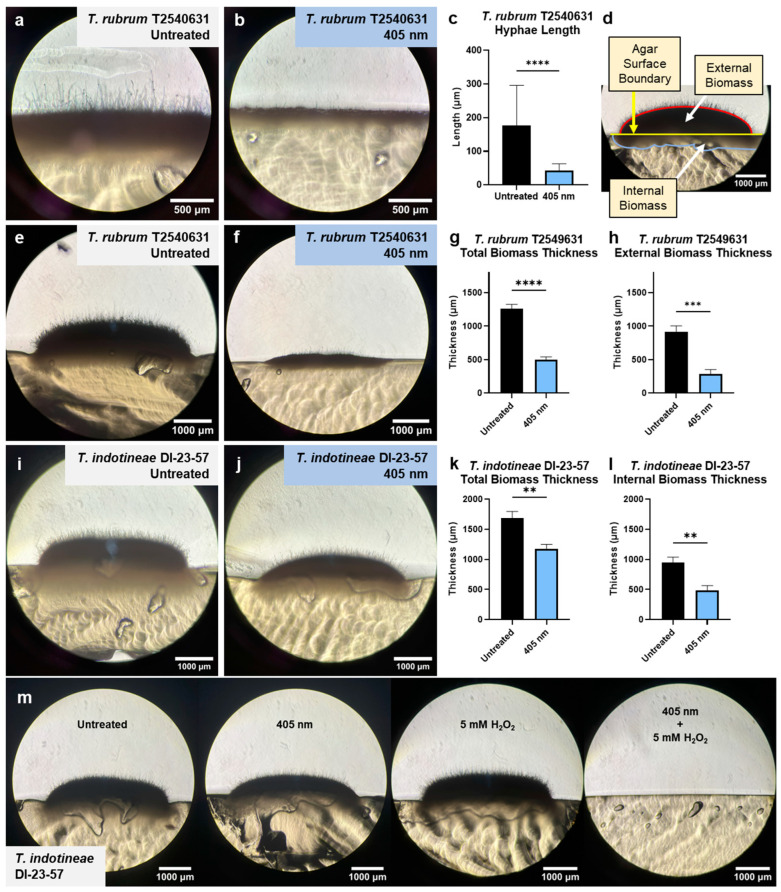
Blue-light exposure suppresses hyphal and biomass formation within dermatophyte strains. Brightfield imaging of *T. rubrum* T2540631 under (**a**) untreated and (**b**) blue-light-treated conditions reveals a (**c**) significant 76.06% reduction in average hyphal length. When measuring the biomass thickness of dermatophytes, (**d**) the overall thickness of the biomass was divided by the agar surface boundary, creating an external biomass region above the surface boundary and an internal biomass region below the boundary within the agar. Brightfield imaging and measurement of (**e**) untreated and (**f**) light-treated *T. rubrum* reveal that light exposure results in a (**g**) 60.5% reduction in total thickness and a (**h**) 69.27% reduction in external biomass thickness. Additional biomass studies performed on antifungal-resistant *T. indotineae* DI-23-57 under (**i**) untreated and (**j**) light-treated conditions indicate that a significant (**k**) 30.2% reduction in total biomass thickness can be observed following light treatment. Unlike the susceptible strain, most of the affected reduction in biomass thickness was located within the internal biomass below the agar surface, (**l**) decreasing by 49.28%. In addition, when blue light treatment is combined with low-concentration H_2_O_2_, (**m**) the complete absence of hyphal growth and development from resistant dermatophytes is observed. Statistical significance: ****: *p* < 0.0001, ***: *p* < 0.001, **: *p* < 0.01.

Previous studies on the downstream effects of blue-light exposure on hyphae-forming fungal species like *Candida* reveal an inhibitory effect on hyphal growth and development caused by disruptions to lipid metabolism. To determine whether similar inhibition can be observed within dermatophytes, *T. rubrum* T2549631 was treated with two 60 J/cm^2^ blue-light exposure sessions over the course of 48 h. Once incubation was complete and the resulting biomass formed was bisected and imaged, significant dermatophyte biomass and hyphae could be observed within the untreated sample (Figure 4a). In contrast, no significant hyphal formation could be fully observed in the light-treated samples (Figure 4b). Measurements of individual hyphae (*n* = 50) showed that while hyphae from untreated samples had an average length of 175.7 μm, treatment with light resulted in a hyphal length of only 42.05 μm, representing a 76.06% reduction in the average length (Figure 4c). 

In addition to a reduction in hyphal length, the observation of dermatophytes on potato dextrose agar plates revealed that light treatment induces changes in the overall mycelial biomass formation. Measurement of the overall biomass was performed by dividing the biomass into two regions, split by the horizontal surface of the agar (Figure 4d). Biomass present above the agar surface was considered “external biomass”, while biomass present below the agar surface invading more deeply into the agar itself was considered “internal biomass”. Measurements of overall biomass length were taken at points where the total biomass was thickest.

For susceptible *T. rubrum* T2549631, brightfield images demonstrate a significant reduction in biomass formation following light treatment (Figure 4e,f). The quantification of the lengths of total biomass indicates that while untreated *T. rubrum* was found to have an average biomass thickness of 1264 μm, light treatment reduced the average thickness by 60.5% to 499.2 μm (Figure 4g). While reductions in thickness were found to occur in both the external and internal biomass regions, the most significant reduction from light treatment was observed in the external biomass above the surface, which saw the average thickness decreased by 69.27% from 918.9 μm to 282.3 μm (Figure 4h). When the same type of light treatment was applied to more invasive and antifungal-resistant dermatophyte strains in the form of *T. indotineae* DI-23-57 (Figure 4i,j), the reduction in total biomass thickness was lower than in the susceptible strain but still significant, decreasing by 30.2% from 1689 μm to 1178 μm (Figure 4k). Interestingly, while no significant difference in external biomass thickness was observed, a nearly 49.28% reduction in internal biomass thickness was measured (Figure 4l). 

While blue-light exposure alone was found to be able to inhibit the hyphal invasion and biomass development of dermatophytes, it was unable to suppress growth completely. Based on the previously observed ROS sensitization observed in dermatophytes, the impact of H_2_O_2_ exposure alongside blue-light treatment was explored within the resistant dermatophyte *T. indotineae* DI-23-57. Unlike the previous agar invasion assays, dermatophyte conidia were exposed to only one session of 60 J/cm^2^ light exposure, followed by treatment with low-concentration H_2_O_2_ (Figure 4m). While significant hyphal and biomass growth can be observed in both the untreated and individually treated samples, no growth was observed to develop in the combination-treated group.

## 4. Discussion

As the leading cause of fungal skin infections, dermatophytes are estimated to affect around 25% of the global population [42]. As a result of this high prevalence, heavy usage of conventional dermatophyte antifungal agents has contributed to the emergence of resistant dermatophyte strains like *T. indotineae* [43]. This growing spread highlights the critical need to identify new methods to target and treat dermatophyte infections. As pathogens primarily localized on the surface and within the stratum corneum of the skin, dermatophytes serve as potential targets for phototherapy in a similar manner to other skin infections such as acne [44,45,46]. While blue-light phototherapy has previously been limited in its potential applicability by its poor dermal penetration, simulations have found that 405 to 410 nm blue light is still able to retain around 90% of its fluence rate as it passes through the stratum corneum [47]. As a result, blue light can easily access the dermatophytes potentially present within a cornified skin layer while retaining most of its energy [48,49]. As a result of this ideal surface localization of dermatophytosis, the applicability of blue-light phototherapy on dermatophytes was explored to determine whether similar degrees of catalase inactivation and ROS sensitization could be observed within dermatophytes, similar to the inactivation found in both bacterial and *Candida* fungi strains [31,32]. 

Based on the results, susceptible *Trichophyton* dermatophytes exhibited significant reductions in catalase activity upon exposure to 405 nm blue light, with two different strains of *T. tonsurans* experiencing a roughly 30% reduction in overall catalase activity. While the initial catalase measurements of susceptible *T. rubrum* strains were unable to be effectively obtained, genomic studies of *T. rubrum* have found it to specifically express the catalase genes TERG_01252 and TERG_06053 as a way of not only protecting against oxidative damage but also maintaining the integrity of the cell during host invasion [50]. The increased ROS sensitization observed in blue-light-treated *T. rubrum* indicates a reduction in overall catalase activity, aligning with similar ROS-sensitizing phenomena observed in bacterial and *Candida* strains [31,32]. In addition to susceptible strains, various antifungal-resistant *Trichophyton* strains also appeared to exhibit significant reductions in catalase activity, although significant variation within the catalase activity reduction was observed. While strains like *T. indotineae* DI-23-55 decreased by only 26.49%, *T. indotineae* DI-23-62 decreased by 86.61%. These variations in reduced catalase activity are likely partly due to alterations in catalase expression between strains, as *T. indotineae* DI-23-62 exhibited significantly higher catalase activity than the other strains. These results are consistent with other dermatophyte strains, such as *Microsporum canis*, which has been observed to demonstrate varied catalase expression [51]. Regardless, the reduction in catalase activity observed in all resistant strains following light treatment confirms that blue light can inactivate the catalase present within the conidia of dermatophytes, opening the door for the potential implementation of ROS sensitization treatments and regiments.

Based on the reduction in catalase activity observed following light exposure, blue-light treatment should therefore correspond to increased ROS sensitization due to the loss of catalase. Thus, H_2_O_2_ was used as a ROS source to treat light-exposed dermatophytes and confirm the occurrence of sensitization. While individual treatments with either 60 J/cm^2^ of blue light or low-concentration H_2_O_2_ were found to have no significant impact on overall viability, a combination of the two treatments resulted in an over 99% reduction in the viability of susceptible *T. rubrum* and *T. tonsurans*. These results confirm that the ROS-sensitizing phenomenon induced by light-induced catalase inactivation is present and can be applied to dermatophytes. The complete eradication of susceptible dermatophytes like *T. tonsurans* upon treatment with both light and H_2_O_2_ was further confirmed through a PrestoBlue viability assay, which not only saw the combination treatment fail to generate any significant fluorescence signal representative of metabolic activity but also revealed, through brightfield imaging, that only the combination-treated dermatophytes remained fully within their conidia form during the incubation period. A similar lack of activity-associated fluorescence under the combination treatment was observed in the antifungal-resistant *T. indotineae* strains, indicating that the antifungal-resistance mechanisms present within resistant dermatophytes, such as overexpressed membrane efflux pumps, drug-inactivating enzymes, and antifungal targets, are unable to compensate for the loss of catalase activity [52]. The high degree of sensitivity to H_2_O_2_ aligns with previous studies that found that, despite being catalase-positive organisms, dermatophytes have been found to naturally express low levels of catalase [53]. Due to this low natural expression, the little catalase expressed by dermatophytes therefore plays a critical role in natural antioxidant defenses against not only exogenous sources of ROS like H_2_O_2_ but also the ROS burst produced by neutrophils within the immune system [54,55]. While increased ROS sensitization within light-treated dermatophytes is likely due to the loss of catalase activity, it is important to note that 405 nm blue light does not exclusively target catalase, and that photoreactions from other endogenous molecules may also be contributing to the improved antimicrobial activity of H_2_O_2_ [56]. Previous studies on the antimicrobial activity of blue light alone have found that endogenously expressed chromophores such as porphyrins and flavins can be photoexcited by blue light and act as intracellular ROS producers within pathogens, contributing to the increased sensitivity to ROS sources. Within bacteria, the ROS produced by endogenous chromophores have been found to be able to induce significant downstream effects, including DNA damage, lipid peroxidation, membrane damage, and protein degradation via carbonylation [57]. Based on the reductions in metabolic activity observed within multiple *Trichophyton* species from light alone, it is likely that that the light-treated dermatophytes are indeed affected by the intracellular ROS produced by their endogenous chromophores. However, for the light dosage applied, these downstream effects do not appear to be enough to induce cell death based on the lack of change in viability observed for the light-treated dermatophytes alone. Therefore, as an exogenous ROS source appears to be needed to take full advantage of the ROS-sensitizing effect of blue light, phototherapy treatment offers a potential method of improving the effectiveness of topical applications of H_2_O_2_ and the natural immune system against dermatophytes.

In addition to the ability of catalase inactivation to increase dermatophyte sensitivity to direct sources of ROS, additional tests were performed to determine whether catalase inactivation improved the performance of more indirect sources of ROS. While the literature indicates that traditional dermatophyte antifungal agents like terbinafine can induce mitochondrial ROS production within filamentous fungi, other fungal studies have found that terbinafine can also act as a free radical scavenger and actively suppress the formation and activity of ROS within dermatophytes [38,58]. As a result, terbinafine is unlikely to significantly synergize with or be enhanced by catalase inactivation. Thus, an alternative antifungal agent in the form of amphotericin B was explored instead, which not only is known to produce antimicrobial ROS as a significant part of its mechanism of action but also has already been shown to exhibit increased performance when combined with blue-light exposure against *Candida* fungi [31,40,41]. In addition, the usage of amphotericin B as a potential agent for the treatment of recalcitrant dermatophyte infections has been explored in recent years [59,60,61]. Assays combining light and amphotericin B revealed that significant improvements could be observed against multiple antifungal-resistant strains. In *T. indotineae* DI-23-57 specifically, amphotericin B concentrations that had a minor to no impact on dermatophyte metabolic activity were found to experience roughly 40 to 50% reductions in activity when combined with light-induced catalase inactivation. While this improvement in activity can be attributed to the increased effectiveness of amphotericin B-induced ROS without the presence of catalase, it is also important to acknowledge that additional interactions between light treatment and amphotericin B could contribute to the antifungal agent’s improved performance. Studies on the mechanisms of amphotericin B have revealed that the drug binds to ergosterol and aggregates vertically within the cell membrane to form single and double ion channels, resulting in membrane leakage and intracellular ROS accumulation [62]. When this aspect is coupled with the membrane damage known to be caused by ROS produced by photosensitized endogenous chromophores [57], it is possible that the reduction in membrane integrity caused by light alone may provide an additional mechanism to improve the incorporation of amphotericin B into the membrane, allowing for improved ROS accumulation and coupling well with the loss of catalase induced by light alone. The implementation of catalase inactivation can, therefore, not only improve the performance of amphotericin B but also allow for potentially low effective concentrations of amphotericin B to be used for the treatment of dermatophyte infections, increasing the overall effectiveness and suggesting new potential methods of treating more antifungal-resistant dermatophyte infections. 

While dermatophytes were found to experience the same type of ROS sensitization following blue-light exposure as other bacterial and fungal strains, the filamentous nature of dermatophytes prompted an investigation into the potential hyphae-suppressing effects of blue light alone on dermatophyte invasion, like the inhibition of hyphal development observed in *Candida* strains following light exposure [33]. Models of dermatophyte skin infections have demonstrated that the germination of dermatophyte conidia begins approximately 24 h after initial contact between the conidia and skin, beginning the production of hyphae that invade the epidermal cornified layer of the stratum corneum [7,63]. If this hyphal development could be suppressed, it may be possible to inhibit the further development and invasion of dermatophyte infections, preventing the maturation of conidia produced by the invading dermatophytes within the skin. Utilizing an agar invasion plate to simulate dermatophyte invasion in a surface environment, light treatment was found to significantly inhibit the formation and development of hyphae from dermatophytes, confirming that catalase inactivation can induce the same suppression of hyphal formation as observed within *Candida*. Interestingly, significant reductions in the mycelium biomass were also observed following light treatment, albeit in differing ways between the susceptible and resistant dermatophytes. Within dermatophyte strains, polarized hyphal development and biomass formation were found to occur in two primary orientations, upwards into the air and downwards into the agar itself, producing both external and internal biomass from the mycelium formed by the resulting hyphae. This directional growth in response to surface contact with the agar aligns with previously observed thigmotropic growth, or directional growth in response to a cell’s physical environment, within dermatophytes [64]. For susceptible dermatophytes, polarized hyphal growth appeared to occur primarily away from the agar surface into the air. In contrast, the resistant dermatophyte strain exhibited the opposite response, preferring to invade deeper into the agar. As a result, light treatment was found to primarily suppress biomass formation in the direction of preferred polarized hyphal growth. While the role of tropic hyphal growth in the virulence of pathogens has been generally debated, the literature indicates that fungal pathogens compromised in tropic orientation exhibit reduced mammalian cell invasion capabilities and virulence [65]. The preferred hyphae and mycelium growth of the resistant dermatophyte may, therefore, be an indication of its increased virulence and invasion capabilities. Furthermore, when light treatment was combined with low-concentration sources of ROS like H_2_O_2_, the total and complete suppression of hyphal growth was achieved. While hydrogen peroxide is not actively used against dermatophytes, hydrogen peroxide cream solutions are effective in treating other skin infections, like acne vulgaris and infected skin ulcers [66,67]. The improvement in H_2_O_2_ activity opens the possibility of further exploring the usage of these ROS-based treatments in conjunction with blue-light exposure. Regardless of the increased ROS sensitivity observed by light treatment, the ability of light treatment alone to inhibit hyphal growth and filamentation presents a potential method of further managing and treating existing dermatophyte infections by suppressing their ability to spread and germinate within the skin. While light treatment is unlikely to enhance the direct antifungal activity of terbinafine, the application of light could be used in conjunction with traditional terbinafine treatment as an additive measure to assist in the management and treatment of dermatophyte infections.

Overall, as the leading cause of fungal skin infections around the world, there is a considerable interest in the development of new treatment methods for dermatophyte infections, especially in the wake of their growing capability for resistance development. Within this manuscript, we demonstrate the ability of blue-light phototherapy to increase ROS sensitization and inhibit polarized hyphal development and growth through catalase-inactivating blue light. By utilizing blue light to reduce the virulence and development of dermatophytes, blue-light phototherapy can be potentially established as a non-invasive and non-drug-reliant method of managing dermatophyte infections, opening new avenues for the potential treatment of these incredibly common infections and identifying methods to help minimize or bypass resistance development.

## Data Availability

The original contributions presented in the study are included in the article. Further inquiries can be directed to the corresponding author.
